# IMPACT OF THE TEACHING NURSING HOME ON NURSING STUDENT’S COMPETENCIES AND WILLINGNESS TO WORK IN LONG-TERM CARE

**DOI:** 10.1093/geroni/igad104.3785

**Published:** 2023-12-21

**Authors:** Howard Degenholtz, Jacqueline Dunbar-Jacob, Kim Ratliff, Donna Fick, Chelsea Dickson, Erin Kitt-Lewis, Emily Franke, Kenna Campbell

**Affiliations:** University of Pittsburgh, Pittsburgh, Pennsylvania, United States; University of Pittsburgh, Pittsburgh, Pennsylvania, United States; Wesley Enhanced Living, Media, Pennsylvania, United States; Penn State University, State College, Pennsylvania, United States; Jewish Healthcare Foundation, Pittsburgh, Pennsylvania, United States; The Pennsylvania State University, University Park, Pennsylvania, United States; Jewish Healthcare Association, Pittsburgh, Pennsylvania, United States; University of Pittsburgh, Pittsburgh, Pennsylvania, United States

## Abstract

The Teaching Nursing Home (TNH) is designed to improve the long-term care workforce and improve quality of care in nursing homes. The initiative has multiple components: enhanced clinical rotations for nursing students with partner schools of nursing, implementation of the Institute for Healthcare Improvement Age-Friendly Health System “4M” quality improvement model, and an online learning network. Nursing students at three schools of nursing participated in clinical rotations at four regional nursing homes. The experience was limited to students in one specific course at each school of nursing. At the beginning and end of the Spring 2023 semester, students rated their competence in: patient assessment, collaborating with the care team, gathering clinical information, medication review, eliciting resident values, and health promotion. Students also rated their preferences for working in long-term care and with older adults. Data from 165 responses at the start of semester (January 2023) and 155 responses to the end of semester (April 2023) survey were analyzed. Analysis of student responses found that students self-rated competencies improved in all areas. Prior to their clinical experience, students ranked working in long-term care and with older adults lower than other settings or populations. The rankings were unchanged after their clinical experiences. Qualitative analysis found that most student comments (50%) were about skills, while only 1.5% were about potential careers in nursing homes. These findings suggest that the Teaching Nursing Home Program is meeting the pedagogical goals, however attitudinal shifts may require different strategies.

